#  

**DOI:** 10.1002/ccr3.5967

**Published:** 2022-06-16

In the article entitled “Hyaluronic acid augmentation pharyngoplasty complicated by retropharyngeal abscess and Grisel syndrome: Case report and literature review”^1^ which was previously published in Volume 10 Issue 5 of *Clinical Case Reports*, the authors have updated the content in Acknowledgment section as follows: “All authors would like to include their sincere thanks to Dr. Jihad Nassar, an otolaryngology consultant at King Abdulaziz Medical City—National Guard Hospital in Riyadh, for his help in caring for the patient and his suggestions regarding the manuscript writing.”
